# Recurrence-free survival after curative resection of non-small cell lung cancer between inhalational gas anesthesia and propofol-based total intravenous anesthesia: a multicenter, randomized, clinical trial (GAS TIVA trial): protocol description

**DOI:** 10.1186/s13741-024-00436-1

**Published:** 2024-07-23

**Authors:** Jeayoun Kim, Susie Yoon, In-Kyung Song, Kyuho Lee, Wonjung Hwang, Heezoo Kim, Dong Kyu Lee, Hyun Kyoung Lim, Seong-Hyop Kim, Jong Wha Lee, Boohwi Hong, Randal S. Blank, Alessia Pedoto, Wanda Popescu, Glezinis Theresa, Archer Kilbourne Martin, Mathew Patteril, Atipong Pathanasethpong, Yada Thongsuk, Tanatporn Pisitpitayasaree, Aijie Huang, Hui Yu, Poonam Malhotra Kapoor, Kyunga Kim, Sang Ah Chi, Hyun Joo Ahn

**Affiliations:** 1 Department of Anesthesiology and Pain Medicine, Samsung Medical Center, Sungkyunkwan University School of Medicine, Seoul, Republic of Korea; 2 Department of Anesthesiology and Pain Medicine, Seoul National University Hospital, Seoul National University College of Medicine, Seoul, Republic of Korea; 3 Department of Anesthesiology and Pain Medicine, Laboratory for Cardiovascular Dynamics Asan Medical Center, University of Ulsan College of Medicine, Seoul, Republic of Korea; 4 Department of Anesthesiology and Pain Medicine, Severance Hospital, Yonsei University College of Medicine, Seoul, Republic of Korea; 5 Department of Anesthesiology and Pain Medicine, College of Medicine, Seoul St. Mary’s Hospital, The Catholic University of Korea, Seoul, Republic of Korea; 6 Department of Anesthesiology and Pain Medicine, Korea University Guro Hospital, Korea University College of Medicine, Seoul, Republic of Korea; 7 Department of Anesthesiology and Pain Medicine, Dongguk University Ilsan Hospital, Seoul, Republic of Korea; 8 Department of Anesthesiology and Pain Medicine, Inha University Hospital, Incheon, Republic of Korea; 9 Department of Anesthesiology and Pain Medicine, Konkuk University Medical Center, Konkuk University College of Medicine, Seoul, Republic of Korea; 10 Department of Anesthesiology and Pain Medicine, Ewha Womans University Medical Center, Ewha Womans University College of Medicine, Seoul, Republic of Korea; 11 Department of Anesthesiology and Pain Medicine, Chungnam National University Hospital, Chungnam National University College of Medicine, Daejeon, Republic of Korea; 12 Department of Anesthesiology, University of Virginia Health System, Charlottesville, VA USA; 13 Department of Anesthesiology and Pain Medicine, Memorial Sloan Kettering Cancer Center, New York, USA; 14 Department of Anesthesiology and Pain Medicine, Yale School of Medicine, New Haven, CT USA; 15 Department of Anesthesiology and Perioperative Medicine, University of Pittsburgh School of Medicine, Pittsburgh, USA; 16 Department of Anesthesiology and Pain Medicine, Mayo Clinic, Jacksonville, FL USA; 17 Department of Anesthesia and Pain Medicine, University Hospitals of Coventry and Warwickshire, Coventry, UK; 18 Warwick Medical School, Coventry, UK; 19 Department of Anesthesiology and Pain Medicine, Khon Kaen University, Khon Kaen, Thailand; 20 Faculty of Medicine, Department of Anesthesiology, King Chulalongkorn Memorial Hospital, Chulalongkorn University, Bangkok, Thailand; 21 Department of Anesthesia and Pain Medicine, Yuhuangding Hospital Affiliated to Qingdao University, Shandong, China; 22 Department of Anesthesiology, Institute of Geriatric Medicine, Beijing Hospital, National Center of Gerontology, Chinese Academy of Medical Science, Beijing, China; 23 Department of Anesthesia and Critical Care, All India Institute of Medical Sciences, New Delhi, India; 24 Biomedical Statistics Center, Data Science Research Institute, Research Institute for Future Medicine, Samsung Medical Center, Seoul, Republic of Korea

**Keywords:** Anesthesia, Desflurane, Inhalational anesthesia, Isoflurane, Lung Neoplasm, Metastasis, Non-small cell lung cancer, Propofol, Recurrence, Sevoflurane, Surgery

## Abstract

**Background:**

Surgery is the primary treatment for non-small cell lung cancer (NSCLC), but microscopic residual disease may be unavoidable. Preclinical studies have shown that volatile anesthetics might suppress host immunity and promote a pro-malignant environment that supports cancer cell proliferation, migration, and angiogenesis, whereas propofol may preserve cell-mediated immunity and inhibit tumor angiogenesis. However, clinical evidence that propofol-based total intravenous anesthesia (TIVA) can reduce tumor recurrence after curative resection remains inconsistent due to the retrospective observational nature of previous studies. Therefore, we will test the hypothesis that the recurrence-free survival (RFS) after curative resection of NSCLC is higher in patients who received TIVA than volatile anesthetics (GAS) in this multicenter randomized trial.

**Methods:**

This double-blind, randomized trial will enroll patients at 22 international sites, subject to study registration, institutional review board approval, and patient written informed consent. Eligible patients are adult patients undergoing lung resection surgery with curative intent for NSCLC. Exclusion criteria will be contraindications to study drugs, American Society of Anesthesiologists physical status IV or higher, or preexisting distant metastasis or malignant tumor in other organs. At each study site, enrolled subjects will be randomly allocated into the TIVA and GAS groups with a 1:1 ratio. This pragmatic trial does not standardize any aspect of patient care. However, potential confounders will be balanced between the study arms. The primary outcome will be RFS. Secondary outcomes will be overall survival and complications within postoperative 7 days. Enrollment of 5384 patients will provide 80% power to detect a 3% treatment effect (hazard ratio of 0.83) at alpha 0.05 for RFS at 3 years.

**Discussion:**

Confirmation of the study hypothesis would demonstrate that a relatively minor and low-cost alteration in anesthetic management has the potential to reduce cancer recurrence risk in NSCLC, an ultimately fatal complication. Rejection of the hypothesis would end the ongoing debate about the relationship between cancer recurrence and anesthetic management.

**Trial registration:**

The study protocol was prospectively registered at the Clinical trials (https://clinicaltrials.gov, NCT06330038, principal investigator: Hyun Joo Ahn; date of first public release: March 25, 2024) before the recruitment of the first participant.

## Background

Lung cancer is the second most diagnosed cancer worldwide and the leading cause of cancer death in men and women combined (Sung et al. 2021). Non-small cell lung cancer (NSCLC) accounts for 80–85% of the total global lung cancer burden (Thai et al. 2021). The standard treatment for stage I, II, and some stage IIIA NSCLC is surgical resection. Despite advances in radiotherapy, chemotherapy, and molecular targeted and immunotherapy, 30–75% of NSCLC patients who undergo curative surgery develop recurrence, which carries a high risk of mortality (Taylor et al. 2012; Yun et al. 2022).

Even with the best technique, a fraction of cancer cells might remain due to incomplete resection margins or iatrogenic “seeding” of tumor cells into the lymphatic and blood streams with surgical manipulation. Some patients may already harbor micrometastases and scattered tumor cells at the time of surgery (Denis et al. 1997; Eschwege et al. 1995; Foss et al. 1966; Hiller et al. 2018a; Horowitz et al. 2015; Vona et al. 2000). Whether this minimal residual disease leads to clinical metastases depends largely on the balance between the ability of the tumor to seed, proliferate, and promote angiogenesis and the activity of anti-metastatic immunity as a host defense (Holmgren et al. 1995; Shakhar and Ben-Eliyahu 2003; Smyth et al. 2001).

There is growing evidence that perioperative factors play a critical role in promoting cancer recurrence and metastasis (Horowitz et al. 2015), and that the anesthetic modality used during surgery can have a significant impact on residual or circulating tumor cells, and the patient’s immune response (Bar-Yosef et al. 2001; Hiller et al. 2018a; Kim and Reviews 2017; Sacerdote et al. 2000; Schlagenhauff et al. 2000; Wall et al. 2019; Yeager et al. 1995).

Anesthetics have the potential to impair numerous components of the host response in animal studies, including neutrophil, macrophage, dendritic cell, T cell, and NK-cell functions (Brand et al. 1997; Markovic et al. 1993; Melamed et al. 2003; Shapiro et al. 1981).

However, anesthetic medication types may have disparate effects on cancer recurrence (Melamed et al. 2003). Preclinical and biomarker studies have shown that volatile anesthetics might promote immunosuppression and create a pro-malignant environment including upregulation of hypoxia-inducible factor that supports cancer cell proliferation, migration, and angiogenesis, whereas propofol may preserve cell-mediated immunity and inhibit tumor angiogenesis (Buckley et al. 2014; Cho et al. 2017; Hiller et al. 2018a; Hiller et al. 2018a; Iwasaki et al. 2016; Jaura et al. 2014; Kim and Reviews 2017; Looney et al. 2010; Markovic-Bozic et al. 2016; Melamed et al. 2003; Tavare et al. 2012; Wall et al. 2019).

Despite the availability of biologically plausible explanations, clinical evidence that propofol-based total intravenous anesthesia (TIVA) can reduce tumor recurrence after curative resection remains inconsistent (Chang et al. 2021; Enlund et al. 2022; Hasselager et al. 2021; Hovaguimian et al. 2021; Jun et al. 2017; Lai et al. 2019; Lee et al. 2016; Makito et al. 2020; Wigmore and Farquhar-Smith 2016; Yoo et al. 2019; Yoon et al. 2022; Zheng et al. 2018), mainly because previously conducted studies have been retrospective or observational in nature. Given the risk of residual confounding inherent in observational studies, randomized trials are required to evaluate the influence of anesthetics on oncologic outcomes.

In this multinational, multicenter clinical trial, we will test the hypothesis that local or metastatic recurrence after curative resection of NSCLC is lower in patients randomized to propofol-based total intravenous anesthesia (TIVA group) than in patients randomized to volatile anesthesia (GAS group). Secondary hypotheses are that overall survival (OS) is increased when patients receive TIVA rather than GAS anesthesia and that postoperative complication rates are not different between the TIVA and GAS anesthesia groups.

In this report, we outline our approach and study protocol to establish an a priori record of the proposed study methods and endpoints.

## Methods

### Study design

This pragmatic, randomized, double-blind, multicenter, and multinational study will be approved by the Regional Ethics Committee in 22 international sites, and informed consent will be obtained from all participants. The steering committee of the study developed the protocol and will manage the conduct of the trial, and obtain and manage study data. The study is coordinated by the Samsung Medical Center (SMC), Sungkyunkwan University, Seoul, Korea, and is registered at ClinicalTrials.gov (#NCT06330038). This clinical trial will be investigator-initiated and non-commercial. The trial will be done in accordance with Good Clinical Practice guidelines, the principles of the Declaration of Helsinki, and relevant regulatory requirements.

### Outcomes

#### Primary and secondary outcomes

The primary outcome of this study is recurrence-free survival (RFS) after curative resection of NSCLC. The observed time-to-recurrence is defined as the duration from the date of surgery to the earliest date between the recurrence/metastasis date and the last follow-up date due to death from any cause, any uncontrollable factor during the study, or the end of the study.

Secondary survival outcomes are locoregional RFS (LRFS), metastasis-free survival (MFS), overall survival (OS), and cancer-specific survival (CSS). The observed time-to-events for LRFS and MFS are defined similarly to that of RFS. The observed time-to-event for OS is defined as the duration from the date of surgery to the earliest date among the date of all-cause death and the last follow-up date due to any uncontrollable factor during the study, or the end of the study. The observed time-to-event for CSS is defined as the duration from the date of surgery to the earliest date among the date of cancer-specific death, and the last follow-up date due to death from any cause other than cancer-specific death, any uncontrollable factor during the study, or the end of the study. Other secondary outcomes are postoperative complications. The postoperative complication is a composite outcome defined as any complication occurring within 7 days post-surgery or at discharge if earlier. The applicable complications are defined by the Society of Thoracic Surgeons (STS) general thoracic surgery databases (Crabtree et al. 2018), and the Clavien-Dindo classification (Wong, Oliver, and Moonesinghe 2017). The postoperative complication is a reasonable surrogate for an immediate postoperative outcome, which we believe will be similar between study groups based on Pasin et al.’s meta-analysis of short-term mortality after propofol vs. volatile anesthesia (95 studies, *n* = 9806 total patients) (Pasin et al. 2015).

#### Safety outcomes

The anesthetics used in this study are routinely administered during general anesthesia and comprise the vast majority of general anesthetics used worldwide. Therefore, our study design does not increase risk compared to standard anesthesia practice except for the risk of randomization. We do not expect any obvious safety outcomes that we believe are due to the intervention.

### Setting and population

**Fig. 1:**
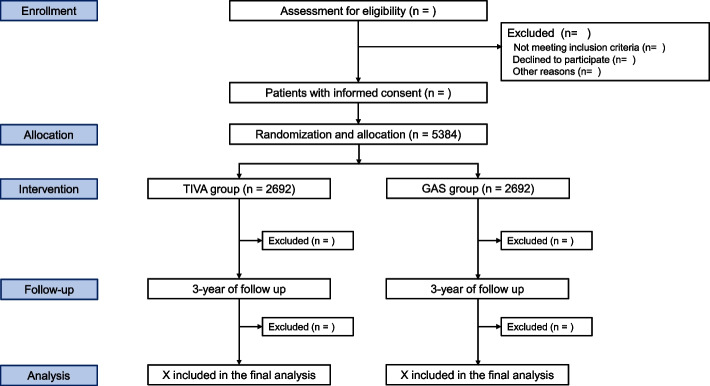
The Consolidated Standards of Reporting Trials flow diagram. TIVA; total intravenous anesthesia, GAS; inhalational anesthesia

We plan a randomized controlled trial at 22 international sites (hospitals in Korea, the USA, Thailand, China, the UK, and India). Screening can be performed either in the outpatient unit or in the ward. The main investigator at each participating site will be responsible for the screening of all adult patients who are scheduled for elective lung resection surgery with curative intent for NSCLC. A screening log will be compiled and will include all screened patients, irrespective of whether or not they are eligible for inclusion. Patients who meet all study criteria will be contacted by the main investigator to obtain written informed consent. A total of 5384 subjects will be enrolled from multiple study sites. Adult patients scheduled for curative resection of NSCLC will be randomized to either the TIVA or GAS group (Fig. 1).

Our inclusion criteria are (1) age 19 years old or older, (2) American Society of Anesthesiologists physical status (ASA) I‒III, (3) The Eastern Cooperative Oncology Group (ECOG) performance status 0–2, (4) Lung resection surgery (segmentectomy, lobectomy, bilobectomy, pneumonectomy; video-assisted, robot-assisted, or open) with curative intent for NSCLC (clinical Tumor, Node, Metastasis (TNM) stage I–IIIA).

Exclusion criteria will be (1) distant metastasis or malignant tumor in other organs as manifested in diagnostic tests such as CT or PET scan, and not in long-term remission according to the attending surgeon, (2) severe neurologic conditions, (3) severe hepatic disease (Child–Pugh classification C), (4) renal failure requiring renal replacement therapy, (5) history of anesthesia and/or surgery within 1 year, (6) previous surgery due to lung cancer (except diagnostic biopsies), (7) contraindications to any study medication (history of allergy, hypersensitivity reaction, or any other contraindication), (8) planned joint extrapulmonary procedure, (9) surgery under cardiopulmonary bypass or extracorporeal membrane oxygenation, (10) planned postoperative sedation, (11) pregnancy, or lactation, (12) patient refusal.

Criteria for exit (drop out) from the trial are (1) patient withdrawal and (2) surgery cancelation. A Consolidated Standards of Reporting Trials (CONSORT) flow diagram will reveal the flow of study participants (Fig. 1).

### Randomization

A stratified block randomization with a block size of 4 will be used to allocate subjects into two intervention groups (TIVA and GAS) with a 1:1 ratio. A computer-generated assignment sequence will be stratified according to the participating site and clinical cancer stage, using a web-based automated randomization system (www.gastiva.org). This system is centralized, password-protected, and encrypted. At each participating site, random allocation will be done according to the assignment sequence before induction of anesthesia.

### Blinding

The group designation will be blinded to the following individuals: (1) patients: patients will not be informed of the randomization assignment and will receive general anesthesia during the intervention, (2) surgeons: the anesthesia process will be initiated prior to the surgeon’s entry into the operating room. This is followed by the placement of opaque drapes that effectively prevent the surgeon from observing any details of the anesthesia technique. The opaque drapes remain in place from the induction of anesthesia throughout the entire surgical procedure, (3) attending anesthesiologists: anesthesiologists in the operating room cannot be blinded to randomization assignment but are not involved in the study enrollment or assessment of the results, (4) each site will have at least two participating investigators (main investigator and outcome assessor). The main investigator will be entering patient information into an electronic case report form (eCRF), being aware of the allocated group, and collecting intraoperative data. The outcome assessor will remain blinded to the allocated group and evaluate the postoperative outcomes. All data will be uploaded on web-based eCRF of www.gastiva.org and group allocation will be opened only when all the collected data are ready for statistical analysis.

### Protocol

The TIVA group will receive propofol for both induction and maintenance of general anesthesia. The GAS group will receive one or more volatile anesthetics (sevoflurane, desflurane, or isoflurane) for induction and maintenance of anesthesia during the surgery. For the GAS group, propofol, midazolam, remimazolam, etomidate, or ketamine can be used with inhalation agents as co-induction agents at the discretion of the attending anesthesiologist. There are no protocol-specific restrictions for concurrent medication in this pragmatic study. In both groups, adjuvant drugs, such as opioids, ketamine, lidocaine, dexmedetomidine, and neuromuscular blocking agents will be administered according to standard institutional procedures at each site, as is postoperative analgesia including neuraxial or regional blocks.

All participating patients, regardless of the study arm into which they are randomized, will be monitored and managed following general standard-of-care practices aimed at maintaining optimal conditions. Both intraoperative and postoperative management (unrelated to anesthetic management) will be decided by the attending physicians, following the established protocols at each center. However, in order to ensure a high standard of anesthetic management, several common strategies have been established: before surgery, the surgical procedure (video- or robot-assisted thoracic surgery or thoracotomy) will be chosen by the attending thoracic surgeon at each center based on clinical features including patient age, comorbid disease, pulmonary function, and tumor characteristics. Appropriate antibiotic prophylaxis will be administered, and standard pharmacological prevention of postoperative nausea and vomiting will be utilized per institutional practice.

During surgery, intraoperative monitoring will include an electrocardiogram, pulse oximetry, capnography, temperature monitoring, anesthetic depth analysis (bispectral index or patient state index), neuromuscular blockade (with a train of four), and blood pressure measurements. Airway management will follow the administration of a neuromuscular blocking agent. The choice of lung isolation device will be at the discretion of the attending anesthesiologist; it is anticipated that most cases will be performed using either a double-lumen endotracheal tube or a bronchial blocker. The device position will be confirmed by fiberoptic bronchoscopy. Anesthesia will be maintained with either propofol or volatile anesthetics based on randomization; opioids, neuromuscular blocking agents, and other adjuncts will be used at the discretion of the anesthesiologist. Propofol and inhalational anesthetics will be titrated to maintain a bispectral index of 40–60 or a patient state index of 30–50 during surgery. Protective ventilation is conducted after the alveolar recruitment maneuver. Mechanical ventilation is maintained with tidal volume (TV) 6–8 mL per kg of predicted body weight (PBW) and positive end-expiratory pressure (PEEP) 5–15 cm H_2_O during two-lung ventilation and TV 4–6 mL per kg of PBW and PEEP 5–15 cm H_2_O during one-lung ventilation, with I:E ratio of 1:1.5–1:2.5 and the ventilation rate is adjusted in the range of 10 to 20 beats per minute to maintain end-tidal carbon dioxide between 35 and 45 mm Hg. The maintenance fluid is the crystalloid solution, infused at a rate of 2 to 5 mL per kg per hour (Lohser 2008). Leukocyte-depleted allogeneic blood will be administered only as necessary to maintain the prospectively determined target hemoglobin. Because hypothermia impairs immune function (Kurz et al. 1996), we will keep patients normothermic—a distal esophageal temperature ≥ 36 °C—with forced-air warming. Tracheal extubation will be at the completion of surgery if postoperative ventilator support is not required. If the patient is returned to the operating room for reasons such as bleeding control, the same anesthetic should be used.

Postoperative analgesia will be achieved based on institutional practice, patient-specific contraindications, and anesthesiologist and surgeon preference. It is anticipated that analgesia will be accomplished based on combinations of neuraxial techniques, regional blocks, and/or intravenous analgesic medications. After approximately 24 h, it is anticipated that patients will be transitioned to primarily non-opioid-regimens (e.g., acetaminophen, tramadol, non-steroidal anti-inflammatory analgesics); however, oral opioids will also be permitted as needed. Maintenance fluid will be administered to maintain a net fluid balance of 0 postoperatively. Ambulation will be encouraged postoperatively as will physiotherapies based on institutional practice (e.g., deep-breathing exercises, incentive spirometry, and chest physiotherapy by physiotherapists and assigned nurses during the intensive care unit and ward stay). Oxygen will be supplied via face mask, nasal prong, continuous positive airway pressure, non-invasive positive pressure breathing, or high flow nasal oxygen supply in patients with SpO_2_ < 90% postoperatively.

### Measurements

Demographic characteristics, details of general anesthesia, drug use including opioid consumption, surgical procedure, and postoperative pain management protocol will be recorded in the eCRF (Bilfinger et al. 1998; Cronin-Fenton et al. 2015; Flores et al. 1996; Freier and Fuchs 1994; Jaeger et al. 1998; Wigmore and Farquhar-Smith 2016; Yeager et al. 2002).

We will also record prognostic factors related to the risk of NSCLC recurrence, including cancer stage, cell type, tumor size, invasion of blood vessels/lymphatics/perineural/visceral pleura, tumor necrosis, and genetic mutation status, based on pathology reports. We will also record whether resection margins are clear of tumor and whether preoperative or postoperative adjuvant therapy was used. For staging, we will use the 8th edition of TNM definitions in lung cancer (2017) which is issued by the International Association for the Study of Lung Cancer. Follow-up of patient records (tumor-specific variables, such as pathologic cancer stage, any metastasis, and different prognostic markers (gene expression) will be extracted by record-linkage, combining the unique individual number with each hospital or national registry at follow-up. If this is not available, patients and their healthcare providers will be contacted for the information.

Cancer recurrence and all-cause mortality will be evaluated by tracking patients at 3 years after surgery. The patient’s medical chart will be reviewed to confirm recurrence status and to obtain details of recurrence if there is one. We will also obtain the results of the chest computed tomography that is performed routinely in these patients. Biopsy results will be obtained whenever possible. The site of initial recurrence will be determined. If patients are lost and their cancer prognostic or survival data are unavailable from the hospital system in participating sites, the investigators will contact the patients or their guardians directly by phone. Cases of apparent recurrence are adjudicated at the trial coordinating site (SMC: Adjudication Committee), using all available laboratory and clinical evidence, provided by the investigators who will still be blinded.

OS after surgery can be ascertained reliably through various sources including querying the National Death Registry, which is the gold standard for vital status in Korea. Other participating centers will use each country’s corresponding data source. The social security number will also be used to determine mortality for patients who are otherwise completely lost to follow-up if allowed by the IRB in each country. Patients who die without local recurrence or metastatic disease will also be censored at the time of death. All measurement variables are listed in Table 1.

**Table 1:** Perioperative data to be collected

Period	Data
Preoperative data	Surgery date Study center Age Sex Weight Height Body mass index Race ASA Physical Status Smoking status Functional status (The Eastern Cooperative Oncology Group [ECOG] performance status) Unrestricted clear fluid ingestion until 2 h before anesthesia Unrestricted CHO-contained fluid ingestion until 2 h before anesthesia Charlson Comorbidity Index Neoadjuvant therapy Clinical stage (The American Joint Committee on Cancer [AJCC] 8th
Operative data	Operative approach Operation name Duration of surgery Duration of anesthesia Intraoperative fluid volume Intraoperative transfusion Intraoperative use of steroids Intraoperative use of opioid Intraoperative adjuvants (ketamine, dexmedetomidine, lidocaine, Steroids, NSAIDs) Use and type of analgesia
Postoperative data	Pathological stage (AJCC 8th) Cell type Tumor size Resection margin Vascular invasion Lymphatic invasion Perineural invasion Visceral pleural involvement Tumor necrosis Mutation Postoperative adjuvant treatment Date of hospital discharge Date of local recurrence Date of metastasis Date of death Cause of death Postoperative complications (Clavien-Dindo classification, STS general thoracic surgery databases) Additional surgeries during the follow-up period and their reason Date of last follow-up

Follow-up chart review, and contact with patients, families, and caregivers will be conducted by outcome assessors who are strictly blinded to group assignment and intraoperative management; questions that might unblind the outcome assessor will be specifically avoided.

Predefined complications are monitored by medical chart review at 7 days or hospital discharge date whichever comes first and recorded in the eCRF.

In accordance with the principles of Good Clinical Practice (GCP), monitoring of the study is arranged by the Sponsor (SMC, Seoul, Korea). SMC is appointed to monitor this study for the Korean sites and for the central monitoring of the database. For the other international sites, the sponsor appoints a local monitoring organization to perform on-site monitoring activities and regular contacts.

The independent data management of the clinical database is managed by the SMC, based on a specific Data Management Plan (DMP). The eCRF serves as the clinical database for the study. In addition, the SMC is responsible for set-up, support, and management of the eCRF. Data management also includes handling queries to resolve any inconsistencies detected by the quality control procedures. eCRF data are subject to both logical computerized checks and manual validation checks against listings in accordance with the study-specific DMP. All inconsistencies detected during these procedures are resolved through queries, being issued to the investigational site personnel.

### Statistics

#### Sample-size calculations

Sample size was calculated based on RFS, the primary outcome of the trial. We expect an overall 3-year recurrence rate of 20% in GAS group based on previous studies (Taylor et al. 2012) [*N* = 1143, event rate of 10%, 20%, and 35% for Stage I, II, III, respectively] and Yun et al. (Yun et al. 2022) [*N* = 6012, event rate of 13%, 35%, and 60% for Stage I, II, III, respectively]). A Cox regression on the anesthesia group (TIVA group versus GAS group) with a sample size of 4845 observations achieves 80% power at a 0.05 significance level to detect a hazard ratio of 0.83 in the TIVA group compared to the GAS group. This HR corresponds to an absolute risk reduction of 3% in the TIVA group compared to the GAS group. Since the most important predictors of NSCLC survival are patient-related factors and aggravating tumor characteristics (Enlund et al. 2023), we expect only a 3% difference related to the anesthetic regimens. Although a 3% absolute difference between study groups is small, we believe it is clinically significant given the ease and low cost of the anesthetic interventions. Anticipating a 10% dropout rate, 5384 subjects should be enrolled to obtain a final sample size of 4845 subjects (in each group, 2692 subjects) (Table 2). No adjustment is made on other covariates in Cox regression, under the assumption that there is no collinearity between the anesthesia group and covariates. As no study has yet examined the effect of the TIVA group compared to the GAS group on RFS, we assumed a conservative condition for the correlation structure which leads to maximizing the estimated sample size. Although we will be conducting competitive enrollment, we expect a total of 5384 subjects to be enrolled in SMC:SNU: Other in a ratio of 4:3:3 (SMC; Samsung Medical Center, SNU; Seoul National University).

**Table 2:** The estimated study power using two-sided log-rank tests at a significance level of 0.05 according to different assumed 3-year RFS rates at each arm

Scenario	Hazard ratio	1/hazard ratio	Risk reduction	*N*	N**	*N* per group			
						Total	SMC	SNU	Others
1	0.65	1.54	5%	1132	1260	630	315	126	63
2	0.73	1.37	5%	1533	1704	852	427	170	85
3	0.78	1.29	5%	2010	2236	1118	559	224	111
4	0.81	1.24	5%	2374	2640	1320	660	264	132
5	0.83	1.2	2%	6460	7180	3590	1796	718	358
6	0.83	1.2	3%	4845	5384	2692	1348	538	268
7	0.83	1.2	4%	3876	4307	2154	1078	430	214
8	0.83	1.2	4%	3230	3592	1796	898	360	178
9	0.77	1.3	3%	3097	3444	1722	862	344	172
10	0.77	1.3	4%	2323	2584	1292	648	258	128
11	0.77	1.3	5%	1858	2068	1034	518	206	102
12	0.77	1.3	6%	1549	1724	862	432	172	86

Power of 80%, Alpha of 0.05, *R*^2^ = 0%, *The hazard ratio* = hazard rate in TIVA group/hazard rate in inhalational group, *The risk reduction* = recurrence rate in the inhalational group-recurrence rate in TIVA group. *Software* PASS 2022 Power Analysis and Sample Size Software (2022). NCSS, LLC. Kaysville, UT, USA, ncss.com/software/pass. N** The total number of subjects that should be enrolled in the study in order to obtain *N* evaluable subjects, based on the assumed dropout rate of 10%. *N per group* The number of subjects that should be enrolled in one group. SMC, SNU, and Others are the categories of participating sites

*RFS* recurrence-free survival, *SMC* Samsung Medical Center, *SNU* Seoul National University; *TIVA* total intravenous anesthesia

#### Data analysis

##### Analysis set definition

Two types of analysis sets will be used: modified intention-to-treat (mITT) and per-protocol (PP) sets. The mITT set includes all subjects allocated in the TIVA or inhalation group, except those who are found to violate any important inclusion criterion or to belong to any important exclusion criterion; who do not undergo the allocated anesthesia during the intervention; and whose primary outcome was not measured during the study. The PP set includes subjects who completed the trial in the originally allocated group except those who meet drop-out criteria. The mITT set will be used as our primary analysis set whereas the PP set will be used as an additional analysis set.

##### Analyses of primary outcome

The rates of RFS will be estimated using the Kaplan–Meier method and will be compared between two intervention groups using two-sample log-rank tests. A univariable Cox proportional hazards regression will be conducted to estimate the hazard ratio for recurrence in TIVA versus GAS groups. As this is a large, randomized trial, we expect that enough number of subjects can be accrued without severe unbalance in each level of major confounding factors such as cancer stage. Multivariable analyses will be further performed to account for possible confounding because they usually can increase the precision of the estimated treatment effect and thus add power to the trial (Canner 1991). In the multivariable Cox proportional hazards regression model, the treatment effect will be estimated after adjusting these confounding factors: center, age, sex, ethnic origin, type of surgery, cancer stage, cell type, tumor size, resection margin, invasion of vascular/lymphatic/perineural/visceral pleura, tumor necrosis, pre-and postoperative adjuvant treatment, major gene mutation status, amount of intraoperative opioids, use of regional analgesia and some covariates with *p* value less than 0.1 in the univariable model (Maher et al. 2014; Sessler et al. 2019). Adjusted survival curves will be drawn to present survival probabilities estimated by univariable or multivariable Cox regression models.

Anticipating a potential difference in postoperative care between centers, we expect that there would be a difference in treatment effect (i.e., the hazard ratio for recurrence in the TIVA group versus the GAS group) between centers. Therefore, a stratified analysis or a clustered analysis will be performed to account for the between-center difference. The violation of the proportional hazards assumption will be visually inspected by Schoenfeld residual plots. If there is a severe violation of the proportional hazards assumption, various advanced methods such as accounting for time-varying confounder or time-dependent effects will be adopted. If a sample size allows, we will conduct subgroup analyses, in which subgroups are constructed according to each cancer type, cancer stage, age group, and sex. These analyses may help identify specific subgroups of patients for whom the intervention appears especially helpful or not helpful. For sensitivity analysis, Fine and Gray’s test will be used to compare cumulative incidence curves for RFS if competing risks due to non-cancer deaths exist.

##### Analyses of secondary outcomes

The LRFS, MFS, OS, and CSS will be analyzed using the same analytic methods as described for the primary outcome above. In addition, Gray’s test will be used to compare cumulative incidence curves for cancer-specific death if competing risks exist. For group comparison in the postoperative complication rate, the chi-square test or Fisher’s exact test will be used as appropriate.

##### Other details for analyses

All baseline variables will be descriptively compared between two intervention groups using appropriate summary statistics, such as mean and standard deviation, median and quartiles, or frequency and percent, and also proper testing methods, including two-sample *t*-test or Mann–Whitney test, chi-square test, and Fisher’s exact test. All tests will be two-tailed, and the statistical significance will be declared if *p* value < 0.05. All data analyses will be performed by a statistics analytic team using SPSS software (version 27.0; IBM Corp., Armonk, NY, USA) or R software (version 4.2.2; R Foundation for Statistical Computing, Vienna, Austria) or SAS software (version 9.4; SAS Institute, Cary, NC, USA) according to a pre-established statistical analysis plan.

### Role of the funding source

The funder of the study has no role in study design, data collection, data analysis, data interpretation, writing of the report, or the decision to submit for publication.

## Discussion

Preclinical studies have demonstrated that volatile anesthetics might promote immunosuppression and the development of a pro-malignant environment that supports cancer cell proliferation, migration, and angiogenesis, while propofol preserves cell-mediated immunity and inhibits tumor angiogenesis (Hiller et al. 2018a; Kim and Reviews 2017; Wall et al. 2019).

Despite these biological explanations, clinical evidence that propofol-based TIVA might reduce tumor recurrence and metastasis after curative resection remains uncertain and inconsistent (Chang et al. 2021; Enlund et al. 2022; Enlund et al. 2023; Hasselager et al. 2021; Hovaguimian et al. 2021; Jun et al. 2017; Lai et al. 2019; Lee et al. 2016; Makito et al. 2020; Wigmore et al. 2016; Yoo et al. 2019; Yoon et al. 2022; Zheng et al. 2018), mainly because previous studies have been conducted as retrospective or observational studies..

Recently, a multicenter randomized controlled trial was published on this subject (the Cancer and Anesthesia Study; CAN NCT01975064). Elund et al. (2023) compared sevoflurane and propofol for 5-year OS in breast cancer (*n* = 1670). The numbers who survived at least five years were 773/841 [91.9% (95% CI 90.1–93.8)] in the propofol group and 764/829 [92.2% (90.3–94.0)] in the sevoflurane group [HR 1.03 (0.73–1.44); *P* = 0.875]. Thus, TIVA did not increase OS compared to sevoflurane anesthesia. Instead, increasing age, BMI, ASA classification, and number of comorbidities were associated with the OS together with polypharmacy, smoking, no alcohol use, and more severe oncological characteristics.

Sessler et al. (2019) compared regional (paravertebral block with propofol sedation) vs. general anesthesia (sevoflurane) (*n* = 2132) in patients with breast cancer. Regional anesthesia with propofol sedation did not reduce breast cancer recurrence after potentially curative surgery when compared with general anesthesia with sevoflurane in a median 3-year follow-up [hazard ratio 0.97, 95% CI 0·74–1·28; *p* = 0·84]. The study design permitted supplemental low-dose sevoflurane in the regional group potentially obscuring differences between Propofol and sevoflurane-based anesthetics.

Both previous studies had event rates (OS or RFS) of only 10%; given the relatively large expected differences in outcomes related to the anesthetic regimens ((a 5% difference in OS (Enlund et al. 2023) and a 30% reduction in cancer recurrence (Sessler et al. 2019) in a total of 1670 and 2132 patients, respectively), statistical power may have been inadequate.

Additionally, results of randomized controlled trials conducted for breast cancer may not relate to NSCLC. The potential of anesthetic drugs to modify tumor biology, including local recurrence and metastasis, may differ substantially between cancer types (Tsuchiya et al. 2003). Breast cancer resection procedures tend to be relatively short and commensurately lower exposure to anesthetic drugs. Furthermore, the superficial location of these tumors may facilitate easier surgical management with a lower risk of cancer cell dissemination. Given the substantial evidence for anesthetic influence on cancer recurrence and host immunosuppression, there is a clear need for additional trials to evaluate the potential benefits of propofol-based TIVA in patients undergoing major surgery. Furthermore, evidence-based medicine requires at least two well-conducted randomized trials to influence guidelines.

Currently, one multicenter study in progress is comparing TIVA versus volatile agents for a variety of major cancer surgeries (lobectomy or pneumonectomy, esophagectomy, radical cystectomy, pancreatectomy, partial hepatectomy, hyperthermic intraperitoneal chemotherapy, gastrectomy, cholecystectomy or bile duct resection, *n* = 1804) with the primary outcome of all-cause mortality (ongoing, NCT03034096). The aforementioned CAN trial includes another arm which includes patients undergoing primary resection of colorectal cancer and is also under progress (Enlund et al. 2019). Another upcoming Volatile Anesthesia and Perioperative Outcomes Related to Cancer trial (VAPOR-C trial), is scheduled to be completed in 2025 in patients with lung or colorectal adenocarcinoma. This trial is a 2 × 2 factorial design comparing propofol versus sevoflurane general anesthesia, with or without intravenous (IV) lidocaine (NCT04316013).

A potential weakness of our study is that it is a pragmatic protocol, meaning that all other aspects of anesthesia, besides the choice of anesthetic, may vary among participating sites. However, the pragmatic nature of the protocol also has the potential to increase the external validity and generalizability.

Confirmation of the study hypothesis would demonstrate that a relatively minor and low-cost alteration in anesthetic management has the potential to reduce cancer recurrence risk in NSCLC, an ultimately fatal complication. In addition, implementation would be easily accomplished since TIVA is a routinely used modality familiar to anesthesiologists. Rejection of the hypothesis would end the ongoing debate about the relationship between cancer recurrence and anesthetic management.

## Data Availability

Data sharing including the full protocol, individual participants, and other relevant study data will be considered upon reasonable request. Only with the permission of the Data Review Board of Samsung Medical Center, the anonymized data will be available from the principal investigator (HJA).

## Abbreviations

AJCC: The American Joint Committee on Cancer
AMC: Asan Medical Center
ASA: American Society of Anesthesiologists
CONSORT: A Consolidated Standards of Reporting Trials
CRF: Case report form
DMP: Data management plan
ECOG: The Eastern Cooperative Oncology Group
eCRF: Electronic case report form
GAS: Inhalational gas anesthesia
GCP: Good Clinical Practice
NSCLC: Non-small cell lung cancer
OS: Overall survival
PBW: Predicted body weight
PEEP: Positive end-expiratory pressure
RFS: Recurrence-free survival
SMC: Samsung Medical Center
SNU: Seoul National University
STS: The Society of Thoracic Surgeons
TIVA: Propofol-based total intravenous anesthesia
TNM: Tumor, node, metastasis
TV: Tidal volume
